# A Comparative Study of Sagittal Balance in Patients with Neuromuscular Scoliosis

**DOI:** 10.6061/clinics/2017(08)05

**Published:** 2017-08

**Authors:** Paulo Alvim Borges, Flávio Gerardo Benites Zelada, Thiago Felipe dos Santos Barros, Olavo Biraghi Letaif, Ivan Dias da Rocha, Raphael Martus Marcon, Alexandre Fogaça Cristante, Tarcíso Eloy Pessoa Barros-Filho

**Affiliations:** Laboratorio de Investigacao Medica, Divisao de Cirurgia da Coluna, Instituto de Ortopedia e Traumatologia (IOT), Hospital das Clinicas HCFMUSP, Faculdade de Medicina, Universidade de Sao Paulo, Sao Paulo, SP, BR

**Keywords:** Neuromuscular Diseases, Scoliosis, Spine Deformity, Sagittal Balance, Surgical Correction

## Abstract

**OBJECTIVES:**

Spinopelvic alignment has been associated with improved quality of life in patients with vertebral deformities, and it helps to compensate for imbalances in gait. Although surgical treatment of scoliosis in patients with neuromuscular spinal deformities promotes correction of coronal scoliotic deformities, it remains poorly established whether this results in large changes in sagittal balance parameters in this specific population. The objective of this study is to compare these parameters before and after the current procedure under the hypothesis is that there is no significant modification.

**METHODS:**

Sampling included all records of patients with neuromuscular scoliosis with adequate radiographic records treated at Institute of Orthopedics and Traumatology of Clinics Hospital of University of São Paulo (IOT-HCFMUSP) from January 2009 to December 2013. Parameters analyzed were incidence, sacral inclination, pelvic tilt, lumbar lordosis, thoracic kyphosis, spinosacral angle, spinal inclination and spinopelvic inclination obtained using the iSite-Philips digital display system with Surgimap and a validated method for digital measurements of scoliosis radiographs. Comparison between the pre- and post-operative conditions involved means and standard deviations and the t-test.

**RESULTS:**

Based on 101 medical records only, 16 patients met the inclusion criteria for this study, including 7 males and 9 females, with an age range of 9-20 and a mean age of 12.9±3.06; 14 were diagnosed with cerebral palsy. No significant differences were found between pre and postoperative parameters.

**CONCLUSIONS:**

Despite correction of coronal scoliotic deformity in patients with neuromuscular deformities, there were no changes in spinopelvic alignment parameters in the group studied.

## INTRODUCTION

In recent years, the sagittal balance of the spine has become a subject of growing interest, and its importance in the treatment of spine pathology has become increasingly recognized 1,2.

Among the most commonly evaluated sagittal radiographic parameters, thoracic kyphosis (TK) and lumbar lordosis (LL) are already widely found in the pediatric population 3,4. However, more recently, interest in other spinopelvic parameters has grown in the medical literature, and research into variations in such parameters in different populations has been increasingly conducted 5.

Proper spinopelvic alignment minimizes energy expenditure during walking and maintaining a horizontal gaze 1 and is associated with a better quality of life in patients with vertebral deformities 6,7. Furthermore, proper alignment helps compensate for imbalances in gait 8.

The optimal strategy for correcting sagittal balance, the clinical significance of this correction and the parameters that indicate its use in everyday practice are not well established for patients with neuromuscular diseases, although they have been determined for patients with idiopathic scoliosis 8,9,10.

The goal of the current study was to characterize the changes that occur in spinopelvic sagittal balance after surgery for correction of scoliosis in patients with neuromuscular spinal deformities. We hypothesized that the procedures that are currently performed do not result in major changes in sagittal parameters, even if they significantly promote the correction of coronal scoliotic deformities.

## MATERIALS AND METHODS

All records of patients with scoliosis who were treated at the Institute of Orthopedics and Traumatology of Hospital das Clinicas, University of São Paulo (IOT-HCFMUSP) between January 2009 and December 2013 were retrospectively reviewed. The study was approved by the IOT – HCFMUSP Hospital Institutional Review Board.

Patients who were not diagnosed with neuromuscular scoliosis or who did not have adequate radiographic records for analysis of sagittal balance were excluded.

Appropriate radiographic recording was considered when panoramic radiographies of the spine in the lateral incidences, which could be observed from the C7 vertebra and femoral heads, were available. The radiographs were measured in the sitting position for non-ambulatory patients and in the standing position for the ambulatory patients.

**Figure 1:**
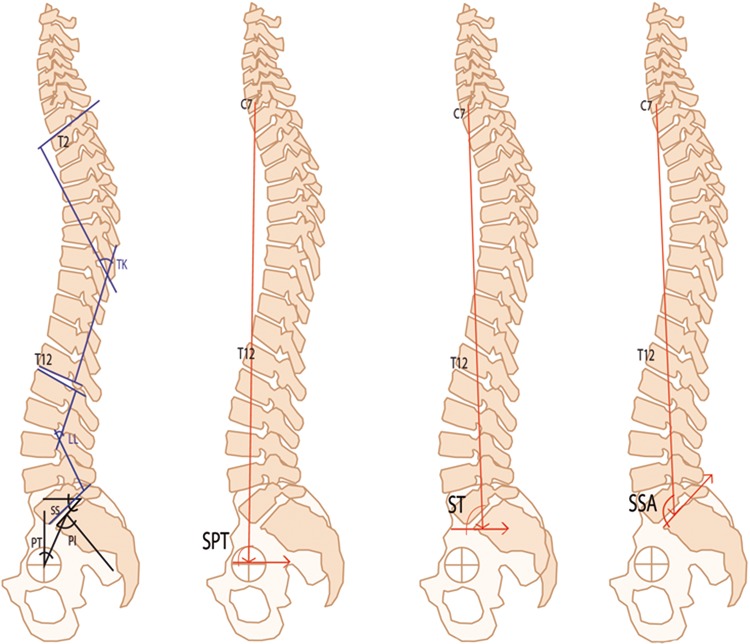
Angle measurements in full-length spine radiographs. Pelvic incidence (PI). Sacral slope (SS). Pelvic tilt (PT). Lumbar lordosis (LL). Thoracic kyphosis (TK). Spinosacral angle (SSA). Spinal tilt (ST). Spinopelvic tilt (SPT).

The radiographic analysis consisted of measuring spinopelvic parameters 1,7,8, TK and LL using Cobb’s method 11. These measurements were performed before and after corrective surgery, and the following parameters were included:

1. Pelvic incidence (PI), defined as the angle formed by lines drawn from the midpoint between the line joining the center of the femoral head and the center point of the upper plateau of S1 and perpendicular to the upper plateau of S1 traced from its middle point (Figure 1).
2. Sacral slope (SS), defined as the angle between a tangential line to the upper S1 plateau and the horizontal plane (Figure 1).
3. Pelvic tilt (PT), defined as the angle formed by lines drawn from the midpoint between the centers of the femoral heads and the midpoint of the S1 plateau and the vertical plumb line to this point (Figure 1).
4. Lumbar lordosis (LL), defined as the angle between tangential lines to the lower plateau of L5 and top of L1 (Figure 1).
5. Thoracic kyphosis (TK), defined as the angle between tangential lines to the lower plateau T12 and superior T2 (Figure 1).
6. Spinosacral angle (SSA), defined as the angle between the sacral plate and a line connecting the centroid of the C7 vertebral body and the midpoint of the sacral plate (Figure 1).
7. Spinal tilt (ST), defined as the angle subtended by a horizontal line and a line from the center of the C7 vertebral body to the center of the upper sacral endplate (Figure 1).
8. Spinopelvic tilt (SPT), defined as the angle formed by a line drawn in the horizontal plane and a line drawn from the center of the vertebral body of C7 to the hip axis, with the middle point of the line joining the center of the femoral head (Figure 1).

The first three angles described above represent spinopelvic parameters in which the pelvic incidence is a constant, intrinsic measure of each individual and can be calculated by the sum of the sacral slope and pelvic inclinations, according to the formula (PI=SS+PT) 1,7. All measurements were calculated in degrees.

All measurements were made using digital, preoperative, full-length spine radiographs from the iSite-Philips (TM) digital display system and with Surgimap^®^ according to a method that has been previously validated for digital measurements of scoliosis radiographs 12.

The generated data were input into Excel^®^ for MAC and analyzed using SPSS 20.0 for MAC. Data analysis was performed using descriptive and inferential statistics. The confidence interval was 95%, and *p*<0.05 was considered statistically significant.

We analyzed the absolute values of the above parameters and compared their pre- and postoperative measurements.

## RESULTS

A total of 101 medical records were assessed. Of these, only 16 patients (7 males and 9 females, age range 9-20, mean age 12.9±3.06) met the inclusion criteria for further study. The main reasons for exclusion were lack of neuromuscular etiology (73 patients) and lack of adequate radiographs for analysis (12 patients). Fourteen patients were diagnosed with cerebral palsy. The Gross Motor Function Classification System – Expanded and Revised (GMFCS - ER) was used for these diagnoses (1 patient was GMFCS I, 6 patients were GMFCS II, 1 patient was GMFCS III, 1 patient was GMFCS IV, and 5 patients were GMFCS 5) 13. Nine patients were ambulatory, and fusion did not extend to the pelvis, while 7 patients were non-ambulatory, and fusion was extended to the pelvis. A senior surgeon who treats more than 50 cases of deformity per year performed all surgeries.

The characteristics of our sample are described below (Table 1).

All data were submitted to Kolmogorov normality testing.

No significant differences were found between any pre- and postoperative measurements, with all *p*-values below 0.05. The results are further described below (Table 2).

## DISCUSSION

Our results show that the mean values of the parameters analyzed agreed with what is expected for such a study population based on the literature 1-5. There were no statistically significant changes between any pre- and postoperative values, as hypothesized.

Different from our findings, La Maida et al. 9 analyzed 76 patients with adolescent idiopathic scoliosis and found a change in sagittal parameters, with increases in PT mean values, concluding that the increase in mean PT value after surgery to be a type of compensatory mechanism for sagittal balance of the spine. This is the first manuscript that specifically addresses changes in sagittal balance in neuromuscular scoliosis.

We believe that the lack of significant changes observed resulted from a number of factors. First, as these parameters are not fully understood, they may be overlooked during surgical planning for correction. Second, the severity of the cases included in this series may have affected the ability to achieve adequate correction (patients with neuromuscular disease tend to present with more severe and rigid curves). Third, given the small sample size of our study, a type 2 error may have occurred. Finally, it is possible that spinopelvic parameters simply remain constant despite surgical correction.

Given the potential for significant improvements in patient quality of life, as demonstrated in the literature 6,7, it is important to study sagittal balance in patients with neuromuscular disease. Although the parameters studied here are already well described in the literature for healthy pediatric populations 3,4, knowledge of their roles in patients with neuromuscular disease is lacking.

It is possible that the lack of such studies in patients with neuromuscular disease results from difficulties in obtaining adequate radiographs and the lack of standardization for radiographic technique. In our study, for example, only 16 of 28 records of the patients with neuromuscular scoliosis (57.14%) were considered suitable for analysis, and the main cause of exclusion was the absence of appropriate radiographs.

In our analysis, only 25% of the included patients were within the range for sagittal balance parameters expected for healthy children. This suggests that patients with neuromuscular disease do not follow the patterns of the healthy population, or perhaps that the parameters used to evaluate such patients should not be the same as those used to evaluate healthy individuals. It is likely that the presence of neuromuscular disease contributes to an imbalance of the sagittal axis in most cases. This inference is in agreement with literature findings that suggest that good sagittal balance minimizes energy expenditure during walking, helps maintain a stable horizon gaze 1 and acts to offset gait imbalances 8, all problems frequently encountered in patients with neuromuscular disease.

We believe that further studies with larger sample sizes are needed to better understand the relevance of these parameters within this specific population. Additionally, the standardization of radiography would be helpful in assessing such parameters moving forward.

No variations were found in the main spinopelvic parameters assessed after corrective surgery in patients with neuromuscular scoliosis in our study. More studies are needed to better understand the impact of the Sagittal Parameters in Neuromuscular Scoliosis and their influence on quality of life in affected patients.

## AUTHOR CONTRIBUTIONS

Borges PA, Zelada FG and Letaif OB provided substantial contributions to the conception, design, data analysis and interpretation, manuscript drafting and revision, and approval of the final version of the manuscript. Barros TF collected the data, revised the manuscript and approved the final version of the manuscript. Rocha ID, Marcon RM, Cristante AF and Barros-Filho TE provided contributions to the data analysis and interpretation, revision and approval of the final version of the manuscript.

## Figures and Tables

**Table 1:** Pre- and postoperative spinopelvic parameters: means and standard deviations (measured in degrees).

		Mean	Standard Deviation
Pelvic Incidence	PI_Pre	43.88	16.899
	PI_Post	47.13	22.704
Sacral Slope	SS_Pre	36.75	21.828
	SS_Post	40.56	9.661
Pelvic Tilt	PT_Pre	7.38	23.804
	PT_Post	7.13	18.048
Lumbar Lordosis	LL_Pre	45.75	30.101
	LL_Post	56.19	11.035
Thoracic Kyphosis	TK_Pre	31.56	27.650
	TK_Post	30.50	13.745
Spinal-sacral Angle	SSA_Pre	123.25	23.188
	SSA_Post	131.94	13.384
Spinal Tilt	ST_Pre	.81	6.685
	ST_Post	.06	6.567
Spinopelvic Tilt	SPT_Pre	86.00	7.099
	SPT_Post	85.50	6.303

**Table 2:** Pre- and postoperative comparison *p*-values.

		*p*-value
Pelvic Incidence	PI_Pre - PI_Post	0.619
Sacral Slope	SS_Pre - SS_Post	0.557
Pelvic Tilt	PT_Pre - PT_Post	0.967
Lumbar Lordosis	LL_Pre - LL_Post	0.202
Thoracic Kyphosis	TK_Pre - TK_Post	0.876
Spinal-sacral Angle	SSA_Pre - SSA_Post	0.168
Spinal Tilt	ST_Pre - ST_Post	0.738
Spino Pelvic Tilt	SPT_Pre - SPT_Post	0.751
